# The Cyberball task in people after obesity surgery: preliminary evaluation of cognitive effects of social inclusion and exclusion with a laboratory task

**DOI:** 10.1007/s40519-021-01297-z

**Published:** 2021-09-12

**Authors:** Paolo Meneguzzo, Elena Tenconi, Enrico Collantoni, Gloria Longobardi, Adele Zappalà, Vincenzo Vindigni, Angela Favaro, Chiara Pavan

**Affiliations:** 1 Department of Neuroscience, University of Padova, Via Giustiniani 2, 35128 Padova, Italy; 2 Padova Neuroscience Center, University of Padova, Padova, Italy; 3 Department of Medicine, University of Padova, Padova, Italy

**Keywords:** Bariatric surgery, Cyberball, Early maladaptive schema, Social cognition, Ostracism, Binge

## Abstract

**Background:**

Social cognition and temperamental and interpretative styles could play a role in the outcome of bariatric surgery. This study aims to assess preliminary evidence about how obesity surgery patients evaluate social inclusion and exclusion through a ball-tossing game called Cyberball, looking at the influence of early maladaptive schemas.

**Methods:**

Thirty-four patients with a history of obesity surgery interventions and 44 controls were recruited for this study. A psychological evaluation was performed before and after the Cyberball task with self-report questionnaires.

**Results:**

In the ostracism condition, significant differences were seen across all the patients’ fundamental psychological needs with less perceived ostracization (*p* = 0.001) even if they recognized less interaction via fewer ball tosses than controls. Moreover, the ostracism paradigm resulted in patients experiencing a higher urge to binge (*p* = 0.010) and a higher urge to restrain (*p* = 0.012) than controls. Looking at differences due to the Cyberball paradigm applied, clear differences emerged only between controls subgroups at the specific self-report scales applied, corroborating the reduced perception of the exclusion. As evidenced by the schema domains, the study found a connection between the impaired limits-schema domain and the drive to binge.

**Conclusion:**

The results show that obesity surgery patients reported different effects of the Cyberball task than controls. Different possible interpretations are discussed, and future directions for studies are exposed, both for the evaluation of social interactions effects and in the assessment of the role of specific cognitive schemas.

**Level of evidence:**

Level III: evidence obtained from well-designed cohort or case–control analytic studies.

## Introduction

Severe obesity is an increasing worldwide epidemic having relevant impacts on people’s daily life, but the efficacy of the treatments is still insufficient [6]. Bariatric surgery is considered one of the most effective treatments, but recent literature has pointed out the lack of knowledge about the elements that could have a positive or a negative effect on its outcome [6]. For example, the psychological effects of weight loss on patients with severe obesity are considered only partially positive because various social or interpersonal difficulties can result in excess skin excess that remains after bodyweight reduction [7, 18]. After a person undergoes bariatric surgery, social and psychological support is sometimes suggested for improving their social interactions and psychological status and reducing the risk of relapse due to excess skin and body image modification [1, 19, 23, 27]. Besides, cognitive aspects could require specific interventions; for example, an individual’s interpretation of interpersonal interactions, whereby they perceive stigma and aloneness, could stem from their childhood and impact the treatment outcome [21, 43].

Early maladaptive schemas (EMSs) are enduring and stable patterns consisting of memories, emotions, cognitions, and bodily sensations that develop in early childhood and affect how a person views themself as an adult in relation to the world [22, 35]. Recent literature has aggregated EMSs into four schema domains: disconnection and rejection; impaired autonomy and performance; excessive responsibility and standards; and impaired limits. These four schema domains comprise the 18 specific EMSs and result from certain self-defeating, core relational patterns learned in childhood and adolescence [4]. These schemas respond to emotional core needs (e.g., love, nurturance, safety, acceptance, and autonomy) being inadequately met by caregivers or significant others, for example. The schemas are repeated in adulthood and are thought to drive a person’s interpersonal dealings (see [34] for more information about EMS). Social isolation, mistrust, and abandonment are three EMSs that have been found to play a role in the psychopathology of people with obesity [37]. Recent studies have identified EMSs as possible obesity maintaining factors. For instance, research has shown that dysfunctional emotion-avoidant strategies could have been embodied during childhood and could drive someone to use eating behaviors to manage their emotions [5, 24]. Psychological treatments of EMS and structured schema therapy treatments have been proven to be clinically relevant interventions in patients with obesity [11, 29]; however, less is known about patients who have already undergone bariatric surgery intervention. For example, no expositive social study has been conducted in the obesity surgery population to evaluate the impact of interpersonal dynamics in cognitive schemas of bariatric patients.

In literature, the effects of social interactions—specifically, interpersonal rejection and inclusion—have been evaluated through Cyberball, which is a virtual ball-toss task that can manipulate people’s experiences by simulating both peer inclusion and ostracism in a standardized way [45, 46]. Several general population studies have demonstrated that Cyberball can authentically simulate social ostracism, causing pain and psychological distress [14, 15]. Studies using the Cyberball task to evaluate patients with obesity have demonstrated the possible roles of shame and social isolation in developing and maintaining an over-eating behavior by showing an increase in food intake after being ostracized [30] as well as the rise of the perceived shame [42]. A recent meta-analysis has shown that people with obesity report higher interpersonal adversity, higher perceived interpersonal stress, and a lower quality of social life; these findings illustrate the need for psychological interventions targeted toward interpersonal sensitivity [2]. However, nothing is known about the responses of obesity surgery patients at the Cyberball task. Since social skills are the basis of interactions between patients and therapists, it could be essential to investigate whether specific patterns could compromise interpersonal behaviors in obesity surgery patients [9].

This study applies the Cyberball paradigm in obesity surgery patients after a stable weight loss to simulate social ostracism and over-inclusion to look for specific connections with the EMS domains. A deeper understanding of bariatric patients' emotional and interpersonal functioning, especially regarding early life experiences, might help to understand better cognitive functioning and responses to different external social scenarios. The first hypothesis is that obesity surgery patients show higher sensitivity and have different cognitive reactions to being ostracized by peers than controls. Our second hypothesis is that specific EMS could be linked to inclusion and exclusion experiences, which would show a potential role as treatment targets in the OS population that might be evaluated with specific studies.

## Methods

### Participants

A group of 34 female subjects with previous severe obesity surgery (OS) was recruited in the outpatient service of the plastic surgery unit of the University Hospital of Padova, Italy. At the time of recruitment, at least 2 years had passed since their bariatric surgery (post-surgery range was 2–10 years), and all patients had maintained a stable weight and were seeking a contouring surgery. The study was proposed to the patients of the contouring service of the hospital following the inclusion/exclusion criteria, and they were voluntary. A group of 44 controls (HC) of matched age, gender, and BMI were recruited from the community by public announcements and without any compensation. The inclusion criteria for both groups were as follows: (1) between 18 and 65 years of age, (2) no severe psychiatric comorbidity, neurological trauma or disorder, or drug addiction, (3) cisgender women. Exclusion criteria for HC were as follows: (1) obesity surgery at any point in their life or (2) extreme lifetime weight loss. A trained psychiatrist evaluated all participants for the inclusion and exclusion criteria. The inclusion of only cisgender women was linked to the reduced number of male patients of the service and to the hormonal and cognitive differences already showed in the literature between males and females during the Cyberball [8, 33]. All the procedures performed in this study were under the Declaration of Helsinki (1964) and were approved by the local ethics committee. All procedures were carried out with adequate understanding and written consent of the subjects.

### Materials

#### Computerized task

Participants were instructed to play a virtual ball-toss game with two other people virtually connected to the game. The Cyberball task was configured as according to previous studies [16, 25], with 30 ball tosses for both conditions: social inclusion and exclusion. The participants received 13% of the tosses (four-ball tosses) during the ostracizing condition and 46% of the ball-tosses in the over-including condition.

#### Questionnaires

Self-report questionnaires were used to evaluate specific psychological features that could influence the Cyberball task results.

Depression was evaluated by the patient health questionnaire (PHQ9), a 9-item self-reported questionnaire that investigates the presence of depression [17]. It is considered a well-established instrument for depression screening. Cronbach’s alpha in the present study was 0.851.

The Young schema questionnaire-short form (YSQ-S3) was used to evaluate EMSs. It is a 90-item self-report questionnaire [36, 47], in which participants are asked to rate a series of statements based on how they felt over the past year. Cronbach’s alpha in the present study was 0.869.

The emotional effects of the Cyberball task were evaluated using the positive and negative affect schedule (PANAS), a 20-item self-reported questionnaire widely used to assess both positive and negative affect and their modifications [38].

The need-threat scale (NTS) is a 21-item questionnaire that indicates ostracism distress [44]. The four dimensions of fundamental psychological needs—belonging, self–esteem, meaningful existence, and control—were used to record feelings of distress or threat. Lower scores on the NTS indicate more significant distress. We added two specific items (“I was ignored” and “I was excluded”) summarized in the manipulation check subscale, as suggested by previous literature, as a measure of the effective achievement of the Cyberball manipulation [32]. Finally, each participant was asked to complete two 10-point Likert scale questionnaires before and after the Cyberball task. These questionnaires rated how strong their urge to binge desire (UTB) or the urge to restrain (UTR) was at that moment.

### Assessment and procedure

All participants were randomly assigned to either the exclusion (i.e., ostracism) or over-inclusion condition with the same methodology used in another study [25]. Over inclusion was selected for this study instead of standard inclusion based on previous studies demonstrating that people with emotional difficulties need unambiguous scenarios in the Cyberball task to elicit specific cognitive, emotional, or behavioral responses [39]. All participants were tested in the same laboratory by the same researchers; the study was conducted in the morning using a 17″ laptop. At the beginning of the session, the participants were evaluated for inclusion or exclusion criteria, and then they completed the pretest self-reported questionnaires. The instructions they received were to participate in an online game with two other female participants sitting in nearby rooms; however, in the Cyberball paradigm, the two other players are computer-generated. At the end of the testing session, the participants completed the need threat scale (NTS) questionnaire, marked their perceived percentage of tosses received on a Likert scale, answered the Likert-scale questions regarding UTB or UTR, and completed the post-test PANAS. Finally, following the recommendation by the international guidelines regarding deception, all participants were debriefed about the deception created by the Cyberball task and informed of the real reason for the task [41].

### Statistical analysis

All the data were analyzed using IBM SPSS Statistics 23.0 (SPSS, Chicago, IL, USA). Different independent *t*-tests were used for the demographic and psychological variables. For instance, the Mann–Whitney test was performed for EMS and estimated ball-tosses received due to their non-parametric distribution. The pre- and post-Cyberball PANAS and eating behavior urges were tested for repeated measures with a general linear model (GLM). The relationships between EMS domains, NTS subscales, and changes in eating urges (calculated both as pre- and post-tests) were tested with correlation analysis using Spearman’s approach. To control for the multi-comparison bias, the Bonferroni correction was used, and only *p* values ≤ 0.013 were considered significant. The effect sizes were calculated with Cohen’s delta. A priori power analysis with data from people with normal and over-weight showed that a sample size of 10 people for each group was sufficient to discriminate inclusion and exclusion with the Cyberball task, with a power of 0.90 and an *α* = 0.05 [42].

## Results

### Characteristics of the participants

No differences in age, BMI, and years of education were seen between the OS and HC groups. Demographic and clinical characteristics are reported in Table 1. However, a significant difference in depression (*t* = 3.311, *p* = 0.002) was found between the two groups.

**Table 1:** Socio‐demographic and clinical characteristics of the study sample

	OS (*N* = 34)			HC (*N* = 44)		*t*	*p*	*d*
	Mean	SD		Mean	SD			
Age (years)	46.09	12.76		41.14	17.15	1.462	0.148	0.327
Weight (Kg) [range min–max]	80.65	16.32		65.98	11.41	4.669	< **0.001**	1.041
	[57.00–138.00]			[45.00–92.00]				
BMI (Kg/m^2^) [range min–max]	28.79	4.95		26.91	3.85	1.888	0.061	0.424
	[19.27–45.06]			[18.42–33.30]				
BMI min (Kg/m^2^)	24.71	5.72		19.43	2.14	5.050	< **0.001**	1.223
BMI max (Kg/m^2^)	46.89	8.52		30.27	4.18	11.312	< **0.001**	2.477
Weight lost after surgery or during lifetime (Kg)	48.79	18.35		2.47	7.62	13.477	< **0.001**	3.297
Education (years)	11.00	3.34		12.60	3.65	1.420	0.100	0.457
PHQ9	7.20	4.15		4.55	2.46	3.311	**0.002**	0.777

*OS* obesity surgery patient, *HC* healthy control, *BMI* body mass index, *SD* standard deviation, *PHQ9* patient health questionnaire, *d* Cohens’ delta effect size, *BMI* min and max: lifetime

Table 2 reports the EMS-domain scores for both populations according to the classification proposed by recent literature [4]. Differences between OS and HC emerged in all the domains: disconnection and rejection (YSQ-DR, *d* = 1.294), impaired autonomy and performance (YSQ-IAP, *d* = 0.906), excessive responsibility and standards (YSQ-ERS, *d* = 0.779), and impaired limits (YSQ-IL, *d* = 0.861).

**Table 2:** The results of the Young schema questionnaire in patients and controls

	OS	HC	Z	*p*
	Mean SD	Mean SD		
**Disconnection and rejection domain** * Self-punitiveness* * Vulnerability to harm* * Mistrust/abuse* * Emotional inhibition* * Pessimism* * Emotional deprivation* * Defectiveness/shame* * Social isolation/alienation*	0.736 (0.528)	0.250 (0.285)	− 4.286	< 0.001
**Impaired autonomy and performance domain** * Dependence/incompetence* * Vulnerability to harm or illness* * Enmeshment/undeveloped self* * Failure to achieve* *Subjugation* * Abandonment/instability* * Insufficient self-control* * Pessimism*	0.617 (0.506)	0.240 (0.304)	− 3.522	< 0.001
**Excessive responsibility and standards** * Subjugation* * Self-punitiveness* * Unrelenting standards* * Enmeshment/undeveloped self* * Subjugation* * Pessimism*	0.597 (0.586)	0.236 (0.294)	− 3.039	0.002
***Impaired limits domain*** * Entitlement/grandiosity* * Insufficient self-control/self-discipline* * Approval seeking*	0.847 (0.673)	0.435 (0.435)	− 2.741	0.006

EMS domains are reported in bold, EMSs that are included in the domain are in italics

*OS* obesity surgery patient, *HC* healthy control, *SD* standard deviation, *Z* standardized coefficient of Mann–Whitney *U* test

### Cyberball results

In the ostracism condition of the Cyberball paradigm, significant differences between the OS and HC subgroups were found for all the fundamental psychological needs, where OS patients reported less perceived ostracization and subjectively fewer ball tosses received, as well as a significantly greater sense of belonging and higher perception of control than the HC group. The OS participants reported a correct estimation of ball passes [14% OS (range 10–20%), 23% HC (range 8–40%), *Z* = − 2.420, *p* = 0.013, *d* = 1.12], but results show that they did not perceive or report an excluded condition. Their reports had mixed results: high belonging and high sense of control with few ball passes and low perception of manipulation.

During the over-inclusion condition, significant differences between the OS and HC groups were found only in the ability to report a correct estimation of the ball toss. OS patients estimated that they received 15% of passes (range 10–20%), whereas HC reported that they received 56% (range 33–86%) of ball passes (*Z* = − 5.502, *p* < 0.001, *d* = 3.89). Looking within populations, OS patients demonstrated less ability to detect ostracism (i.e., no significant difference between ostracism was perceived between OS participants in the ostracism condition versus the over-including condition) even if they were able to estimate the received number of ball tosses correctly in the ostracized condition. Additionally, there were no differences in self-esteem levels in the OS population based on whether they were in the included versus excluded group. However, there was a significant impact on self-esteem in the HC group based on whether they were included or excluded (*t* = − 3.475, *p* = 0.001). See Table 3 for detailed results of the Cyberball conditions’ effects on each group.

**Table 3:** Cyberball exclusion and overinclusion conditions detection in patients and controls

	Ostracizing condition							Over including condition							Ostracism vs. overinclusion			
	OS (*N* = 16)		HC (*N* = 21)		*t*	*p*	*d*	OS (*N* = 18)		HC (*N* = 23)		*t*	*p*	*d*	OS vs. OS		HC vs. HC	
	Mean	SD	Mean	SD				Mean	SD	Mean	SD				*t*	*p*	*t*	*p*
Belonging	17.00	3.54	12.05	3.38	4.324	< **0.001**	1.43	20.33	3.12	21.13	3.49	− 0.759	0.453	0.24	− 2.917	**0.006**	− 8.743	< **0.001**
Self-esteem	13.06	3.41	16.19	3.42	− 2.753	**0.009**	0.92	16.67	5.21	18.91	3.86	− 1.586	0.121	0.49	− 2.409	0.022	− 2.464	**0.012**
Meaningful existence	16.75	4.17	15.86	2.17	0.779	0.444	0.27	19.61	2.93	20.13	2.79	− 0.579	0.566	0.18	− 2.334	0.026	− 5.635	< **0.001**
Control	13.06	3.13	9.57	2.50	3.773	**0.001**	1.23	16.50	3.87	15.35	3.45	1.007	0.320	0.31	− 2.825	**0.008**	− 6.310	< **0.001**
Manipulation check	4.19	2.34	6.57	2.38	− 3.039	**0.005**	1.01	2.78	1.81	2.13	0.63	1.458	0.160	0.48	1.979	0.046	8.298	< **0.001**

Significant *p*-values are in bold

*OS* obesity surgery patient, *HC* healthy control, *SD* standard deviation, *d* Cohens’ *d*

### Emotional and eating urges change due to the Cyberball paradigm

**Fig. 1:**
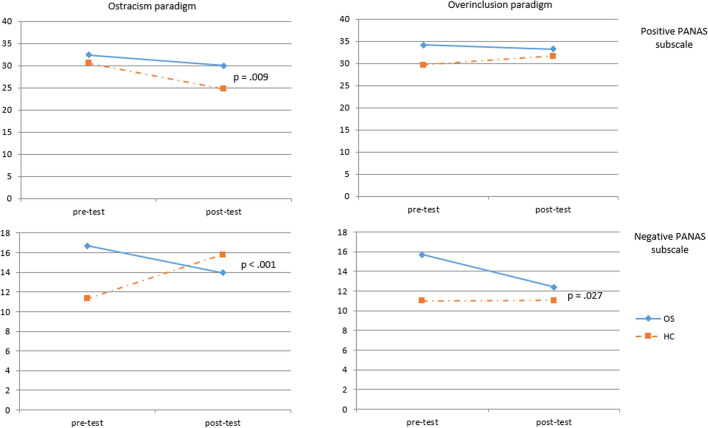
Emotional changes pre-post Cyberball tasks. *OS* obesity surgery patients, *HC* healthy controls

As for the emotional changes, Table 3 and Fig. 1 show reporting of mood changes by both groups. A significant effect of the diagnosis—emotional changes interaction with the GLM analyses for repeated measures was found in the ostracized condition both for the positive subscale (*F* = 7.616, *p* = 0.009, *η*^2^ = 0.192) and for the negative subscale (*F* = 28.204, *p* < 0.001, *η*^2^ = 0.468). In the over-included condition, no significant differences were found between both groups (positive: *F* = 3.228, *p* = 0.082, *η*^2^ = 0.089; negative: *F* = 5.373, *p* = 0.027, *η*^2^ = 0.140).

**Fig. 2:**
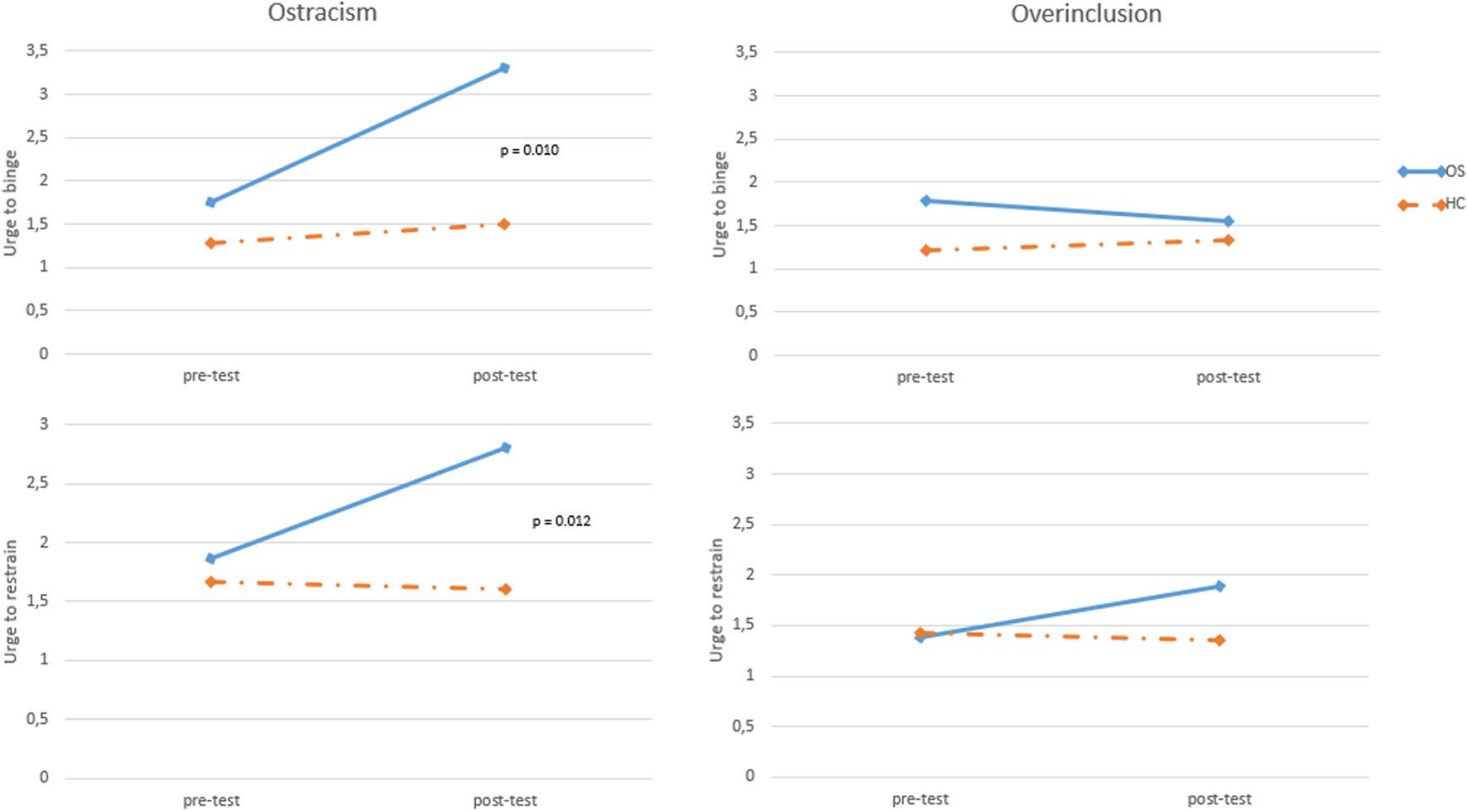
Scores of the urge to binge and the urge to retrain scales. *OS* obesity surgery patients, *HC* healthy controls

With reference to the UTB and UTR scales, the participants showed no differences in the baseline between ostracized subgroups (UTB: *t*(32) = 2.127, *p* = 0.044; UTR: *t*(33) = 0.609, *p* = 0.547) and over-included subgroups (UTB: *t*(33) = 2.161, *p* = 0.041; UTR: *t*(33) = 0.216, *p* = 0.830). After the Cyberball paradigm, the self-reported changes in UTB and UTR were evaluated using a GLM for repeated measures: in the ostracism paradigm, the pre- and post-UTB scores demonstrated a significant interaction time *x* diagnosis (*F* = 7.485, *p* = 0.010, *η*^2^ = 0.190) as did the UTR scores (*F* = 7.056, *p* = 0.012, *η*^2^ = 0.181). In the over included condition, no significant interaction time *x* diagnosis has been found (UTB: *F* = 2.628, *p* = 0.114, *η*^2^ = 0.072; UTR: *F* = 2.678, *p* = 0.111, *η*^2^ = 0.073). The results are shown in Fig. 2.

### Correlation analyses

The correlation analyses for the relationship between the fundamental needs evaluated with the NTS and the EMS domains showed significant results only in the OS patients who were placed in the ostracized condition. Significant relationships were found between NTS-self-esteem and YSQ-ER (*ρ* =  − 0.599, *p* = 0.013), and YSQ-IL (*ρ* =  − 0.652, *p* = 0.006). No significant correlations were found in the HC population in either of the conditions. Upon examining the relationship between schema domains and UTB and UTR, a significant correlation was only found between YSQ-IL and change in UTB scores (*ρ* = 0.735, *p* = 0.011).

## Discussion

The main goal of this study is to investigate how OS patients perceive a social-exclusion situation and their cognitive reaction to this condition in a laboratory task, as well as the influence that specific cognitive and emotional styles, called EMS, have on the perception of specific social situations (e.g., ostracism).

Looking at the baseline differences between our subsamples, as expected, due to the inclusion and exclusion criteria to participate in this study, weight history showed a higher maximum BMI in the OS patients, and the depression evaluation showed higher scores in OS patients than HCs [17]. Finally, as suggested by the literature, the results regarding EMS domains show a greater impact of EMS in the OS population. This finding confirms the possible presence of early dysfunctional cognitive schemas developed from early interpersonal experiences that could influence bariatric patients’ behavior [11].

Our results showed that OS patients correctly reported fewer ball passes and higher ostracism scores in the ostracized condition, but also higher feelings of belonging and control than HC participants, demonstrating a lower awareness of their exclusion from the toss-ball game. Moreover, looking between OS subgroups, the comparison of the psychological needs scores showed less significant differences than controls’ comparison. These might suggest that OS patients could be less accurate in identifying and mentalizing negative situations or emotions, or they might also suggest a possible impairment in the laboratory evaluation of social interactions. Furthermore, the results indicate the possible presence of a cognitive profile secondary to a specific bias for processing harmful interpersonal contact characterized by a detachment from a negative situation, which has been already proposed by previous literature [12, 48]. Moreover, the literature has already demonstrated that people with obesity use emotional suppression as a coping strategy in everyday life [48], and our data confirm this finding by showing that emotional changes are reduced or opposite if compared to matched controls. The OS sample also showed no difference in impact on self-esteem, whether in the exclusion group or inclusion group. This finding could be viewed as a form of detachment from specific social-based negative experiences. But this detachment is not neutral concerning eating behaviors. Indeed, OS patients reported higher levels of drive to binge or restrain from foods, demonstrating that being excluded could affect bariatric patients by inciting a response to focus on food. The effect of food on the improvement of mood levels in patients with obesity and overweight is a well-known phenomenon [20], but the evaluation of the interpersonal dynamics should be included in this model. However, our results could also be linked to the nature of the laboratory task, which could be insufficient to modify a stable psychological profile in OS patients with the ostracized condition. Future studies should evaluate social inclusion/exclusion effects with different methodology and use more immersive environments.

The IL domain is explicitly linked to eating behaviors after social exclusion. This domain represents the lack of internal limits, an inability to form long-term goals, and a lack of responsibility to others, and it has already been linked to addictions and food behaviors [3, 5]. Shame and overeating have already been shown to be emotional and behavioral responses to being excluded in subjects with obesity [30, 42]. The results of this study extend these previous findings to include patients who have had bariatric surgery. This study also shows which cognitive schemas could be implicated. The sense of self and the understanding of interpersonal boundaries could be the specific targets of interventions to improve social skills in OS patients. Moreover, the presence of cognitive schemas that external events could provoke is corroborated by the results of the UTB and UTR scales. Our results show that OS patients present a reaction to stressful social events (like being ostracized) significantly different from the HC peers (even though it was not cognitively perceived), which translated into eating cognitive engagements. Previous studies have already shown that OS patients try to control stressful events with eating concerns or control. For this reason, these schemas might be considered as possible targets of psychotherapy treatments [26, 40]. Indeed, stressful events impact eating behaviors [30], and cognitive and behavioral responses could be targeted as weight-maintaining factors. Our data support this idea because it shows that, even after stable weight loss, an exclusion from social interaction could require more effort for OS patients, even if stricter eating controls are reinforced (e.g., OS patients were able to reduce their weight drastically and to keep it stable to receive contouring intervention).

Finally, the participants’ expectations should be taken into consideration. Niedeggen et al. [28] have shown that belonging, meaningful existence and control in the NTS scale are related to the cognitive expectancy of social involvement. These thoughts and ideas can be described on a continuum ranging from ostracism to inclusion in Cyberball tasks. From this perspective, data from this study could also be interpreted as an expectation by OS patients to be excluded by peers, which could cause the mixed results on the NTS scale after the ostracism paradigm. However, these results could also be interpreted as a resilient aspect of the OS patients to be cognitively altered by non-sufficiently ecological conditions; such is a laboratory task. Our controls have a similar BMI but a different maximum BMI, so they might be exposed to less social exclusion than OS patients. Obesity surgery patients could have developed a defense mechanism from negative social interactions, evidenced by their emotional and cognitive responses, and may need more immersive tasks to be elicited. This aspect of the research could help confirm the need for a global call to action, which has recently been advocated because weight bias could produce pervasive negative attitudes or beliefs, expressed as stereotypes, prejudice, and even open discrimination toward individuals with obesity or overweight [10].

A longitudinal approach and a research-mediated evaluation of schemas could help establish the effects of obesity, bariatric surgery, and weight loss for future research. Furthermore, clinical outcomes from improving specific cognitive schemas could augment already promising treatments focused on managing food cues focused on cognitive approaches [31].

### Strength and limits

In examining the methodology, the suitable match between samples (OC and HC) and the choice to use over-inclusion conditions should be considered a strength of our study because it provides a strong comparison for the ostracism results. These different scenarios have been demonstrated as more acceptable conditions for clinical populations with impaired social and emotional skills (e.g., borderline personality disorder; [13]). Moreover, the inclusion of only cisgender women should be considered a strength of the study due to the different hormonal and cognitive responses gender-driven. Still, it is also a limit because the results are generalizable only to women. A possible limitation involves the scales used due to their self-reported nature, even though they are well-validated measures or the cross sectional nature of the study. Moreover, the Cyberball task could be implemented into virtual reality, with a more immersive environment, that could reinforce our results. Using a cognitive evaluation to determine the participants’ food-related behaviors instead of an authentic assessment of food behaviors after social exclusion or inclusion could also be considered a limitation of this study that may be overcome in future evaluations. Finally, the recruitment of the OS patients with a positive outcome of the surgery and significant weight loss might limit the interpretation of the results. Indeed, patients who failed their weight loss should also be included in future studies for direct comparisons.

## Conclusions

In summary, the OS patients seem to be less able to differentiate social inclusion from exclusion in this laboratory task. Ostracism is shown to have a stronger link to eating cognitive engagement in OS patients than HCs, pointing out a possible cognitive mechanism of interpreting interpersonal functioning that could influence the bariatric outcome due to the eating behaviors that could follow social exclusion. However, more studies are needed to confirm our data and clarify the effect of social exclusion on the psychological profile of OS patients. The data also demonstrate how EMS might play a role in interpreting the interactions between people, possibly revealing a strategy for intervention. In other words, schema results show that impaired limits domain (general lack of responsibility to others, internal limits, future goals, or all three) is correlated to eating urges in the OS population, pointing out a possible direction for future studies.

## What is already known on this subject?

Interpersonal difficulties could have negative effects on obesity surgery outcomes, and psychological support is suggested to improve the outcomes. Early maladaptive schemas are pervasive self-defeating or dysfunctional themes or patterns of memories, emotions, and physical sensations that have negatively affected obesity surgery outcomes, but studies are lacking.

## What this study adds?

This study adds information about cognitive responses of obesity surgery patients after social exclusion or over-inclusion, specifically their inaccuracy to evaluate social exclusion. Specific cognitive schema domains have been pointed out as possible targets of future studies focused on the psychological improvement of the bariatric surgery outcome.

## Data Availability

The datasets generated during and/or analysed during the current study are not publicly available due to ethical restrictions to protect the confidentiality of the participants but are available from the corresponding author on reasonable request.
